# A Robust Visual Tracking Method Based on Reconstruction Patch Transformer Tracking

**DOI:** 10.3390/s22176558

**Published:** 2022-08-31

**Authors:** Hui Chen, Zhenhai Wang, Hongyu Tian, Lutao Yuan, Xing Wang, Peng Leng

**Affiliations:** 1 College of Information Science and Engineering, Linyi University, Linyi 276000, China; 2 School of Physics and Electronic Engineering, Linyi University, Linyi 276005, China; 3 Shandong (Linyi) Modern Agricultural Research Institute, Zhejiang University, Linyi 276000, China

**Keywords:** transformer, cross-attention, CNN, transformer-based tracker

## Abstract

Recently, the transformer model has progressed from the field of visual classification to target tracking. Its primary method replaces the cross-correlation operation in the Siamese tracker. The backbone of the network is still a convolutional neural network (CNN). However, the existing transformer-based tracker simply deforms the features extracted by the CNN into patches and feeds them into the transformer encoder. Each patch contains a single element of the spatial dimension of the extracted features and inputs into the transformer structure to use cross-attention instead of cross-correlation operations. This paper proposes a reconstruction patch strategy which combines the extracted features with multiple elements of the spatial dimension into a new patch. The reconstruction operation has the following advantages: (1) the correlation between adjacent elements combines well, and the features extracted by the CNN are usable for classification and regression; (2) using the performer operation reduces the amount of network computation and the dimension of the patch sent to the transformer, thereby sharply reducing the network parameters and improving the model-tracking speed.

## 1. Introduction

Object tracking as a fundamental computer vision task has widespread applications in multiple fields, such as visual surveillance, human–computer interaction, and augmented reality. Essentially, object tracking obtains the position and shape of a tracked object in a continuous video sequence. Despite considerable efforts in recent years, highly accurate real-time trackers are still challenging to design and implement because of interference from illumination changes, occlusions, severe deformations, background clutter, etc.

In recent years, researchers have used deep learning methods to track objects rather than traditional methods, such as particle filtering by Zhang et al. [1] and correlation filtering [2,3,4,5,6,7,8]. In addition to the advantages of end-to-end learning, deep learning-based tracking has better feature representation than the traditional handcrafted features through convolution output. Bertinetto et al. proposed SiamFC [9], and it has become popular among the tracking methods based on deep learning. The procedure of the Siamese network tracker uses two processed images as the template region in the tracking stage and the search region. The target to be tracked in a given frame is the template region, and the search region is the subsequent frame of the template area frame in the video sequence. After the CNN with shared weights extracts the template region and the search region, the feature map generated by the template region is used as the convolution kernel, and the feature map generated by the search region is used as the input. This input determines the bbox of the tracking target we selected in the search region. Then, the score map generated by this operation is not a strict feature map, and it is easy to lose a lot of semantic information, so it is not conducive to the subsequent classification and regression operations of the network. Using CNN, HOG, and CN, Danelljan et al. [10] also achieved good results in tracking tasks. Vaswani et al. proposed a transformer [11] for the language model structure. Recently, transformers have excelled in visual classification tasks, including image classification [12] and object detection tasks [13,14], and some researchers have introduced a transformer into the object tracking task [15]. However, the existing transformer tracker only reshapes the features extracted by the CNN directly into patches, where each patch contains a single element of the spatial dimension of the extracted feature to be fed into the transformer.

**Figure 1:**
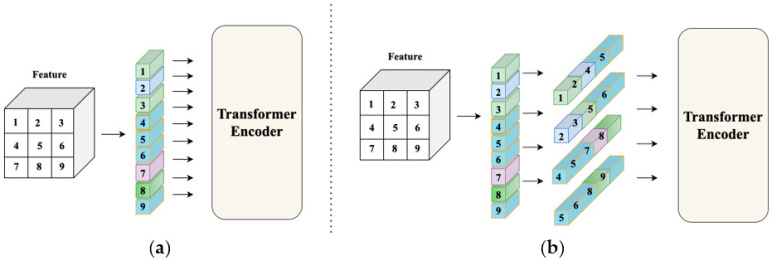
(**a**) Traditional transformer calculations. (**b**) Reconstruction patch’s transformer calculation.

The structure uses cross-attention to replace the cross-correlation operation shown in Figure 1, but forming a patch in this way undoubtedly damages the integrity of the features extracted by the CNN. This paper proposes a method of reconstructing the patch to fully use the integrity of CNN-extracted features and combine adjacent feature element groups into a new patch in the form of a window to maximize the integrity of the target object. Inspired by T2T transformers, this method uses soft segmentation to ensure that it models critical local structures between the adjacent pixels. Unlike T2T, we model the extracted features of the backbone which protects the relative pixel position information for a better tracking effect, as shown in Figure 1. In addition, the repeated use of pixels results in excessive parameters and slow tracking speed. For this reason, we used [16] to solve this problem and finally achieve the effect of real-time tracking.

1. We propose a new transformer architecture in the object-tracking application, reconstructing the per-pixel patch to the window-level patch for attention calculation for visual tracking. We elevate the original pixel-level attention to the new window-level attention. Expanding the pixel-level patch to the window-level patch brings greater precision.
2. The reconstruction patch method uses the patch over-reconstruction method to build a window-level patch. Thus, it uses the features extracted by the backbone to improve the accuracy of window attention and improves FPS by reducing the embedding dimensions and the number of parameters.
3. We reduce the amount of network computation and apply the transformer encoder of the reconstruction network using the method in the performer.

## 2. Related Work

### 2.1. Cross-Correlation-Based Tracking

Since deep learning methods have the advantage of end-to-end learning and the generated features are superior to the traditional handcrafted features, most of them used for object tracking in recent years are Siamese-based. The key to these methods is to use cross-correlation to calculate the similarity map of two pictures to determine the position of the tracked target. The primary operation of SiamFC is to cross-correlate the features generated by the template region and the feature maps generated by the search region. The former is the convolution kernel, and the latter becomes the input to generate the score map. The highest score is the target we need to detect and pass to the scale space to get the location of our target. However, SiamFC can only determine the center of the target, and there is no way to get its size. Therefore, it uses a simple multiscale detection method to obtain the target position, but the accuracy is not high when the number of calculations increases. The SiamRPN method [17] for the object detection task proposed by Ren et al. is Faster-RCNN [18]. It learns its advanced experience and adds the RPN structure based on SiamFC to predict the target, siamRPN. There is a need for multiscale testing so it is not only faster, but also more accurate than SiamFC. Both SiamRPN and SiamFC use simple AlexNet [19] as the backbone of network feature extraction and perform the cross-correlation operation on the extracted features to calculate the score map. To solve the problem of the network backbone, Li et al. proposed SiamRPN++ [20]. They analyzed this technique and practiced using deeper networks in the Siamese network object tracking because of the particularity of object tracking. The network must meet strict translation invariance, but the padding operation will destroy this property. Therefore, SiamRPN++ offsets the target near the centerpoint using a uniformly distributed sampling method, which alleviates the influence of the network because it destroys the strict translation invariance. That is, it eliminates the position bias so that the modern network can perform the tracking. In addition, the method of multilayer feature fusion allows the network to better use them for classification and regression uses the RPN preset anchor to return to the frame. Although this method is very efficient, setting an anchor will not only introduce ambiguous similarity scores, but also require a large amount of data distribution, a priori information that does not fit the purpose of generic tracking. In response to this problem, SiamFC++ [21] classifies and regresses the corresponding map to generate a regression box without presetting anchors. SiamFC++ adds position regression and quality scores based on SiamFC and uses a variety of loss joint training to improve the tracking performance significantly on the original basis. SiamBan [22] and SiamCar [23] are similar to SiamFC++. The difference is that SiamBan and SiamCar use multilayer feature fusion, in which SiamCar creates a new centrality score map to determine the best target centerpoint.

### 2.2. Vision Transformer

The transformer structure proposed by Vaswani et al. uses the long-distance dependence of equivalent words in natural language processing. Devlin et al. [24] made the best proposal. The main structure of a transformer is the attention structure, which takes the sequence as an input and calculates the relevance of each word in the sequence. ViT [25] introduced a transformer into computer vision for the first time. It divides the image to obtain patches that the transformer can receive for the attention operation. Being the first method to introduce the transformer into the field of computer vision, it does not provide ideal results when training on small and medium datasets. However, the performance improves after pretraining on large datasets, and the final result surpasses the mainstream CNN structure network of the year. T2T-ViT [26] are based on ViT and show that simple tokenization of the input image cannot model important local structures (e.g., edges, lines) between the adjacent pixels. This lack leads to the problem of low training sample efficiency, thus designing a method from tokens to tokens. A technique similar to the convolution operation is used to splice the area where the template slides are according to the spatial dimension to form a new token to model the local structure of the image. Our method borrows the idea of T2T-ViT, but it is fundamentally different. We form a new patch for the feature map and perform the attention operation, while T2T-ViT directly generate a token for the attention operation. This article ensures the integrity of the feature map when performing the attention operation, while T2T-ViT model the local structure to ensure integrity between the adjacent elements. In addition to T2T-ViT, compared to ViT, the Swin transformer [27] adopts window-based local self-attention, which can split the input when dealing with large-resolution images or visual tasks with dense output. More windows cause a linear computational complexity suitable for vision tasks with high-resolution input and dense output.

### 2.3. Cross-Attention-Based Tracking

The cross-attention-based tracker is similar to the previous correlation filtering-based tracker network. Transt also uses ResNet [28] as the network backbone as a feature extractor, unlike the previous correlation filtering-based tracker. The correlation operation will cause semantic information loss, resulting in local optimization. Transt uses cross-attention to calculate the similarity between the template region feature and the search region feature. Specifically, it uses a feature fusion network to replace the previous correlation. The feature fusion network contains two modules, ECA and CFA. ECA provides feature enhancement. It obtains the fusion vector by calculating the enhanced template region features and the search region features element by element according to the spatial dimension. Finally, it classifies the fusion vector and performs regression using a fully connected layer.

TrDiMP [29] separates the encoder and the decoder in the transformer into two parallel branches and designs the two branches as a twin-network trace task pipeline. Unlike the original transformer where the encoder section features the backbone-extracted template feature, attention enhances the feature. The search feature, on the other hand, is handled via the decoder. However, as the authors say, TrDiMP does not handle occlusion and out-of-view very well.

## 3. Our Method

**Figure 2:**
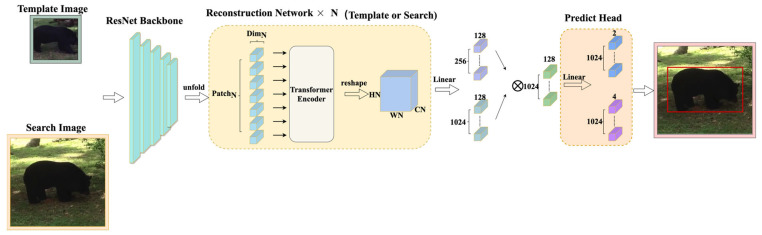
Architecture of our transformer tracking framework. In this figure ×N represents the number of times the network is repeated.

**Figure 3:**
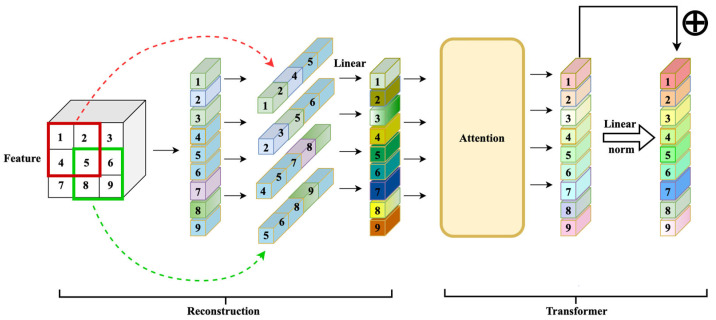
Reconstruction network.

This section introduces the transformer tracker that refactors the patch. Our tracker contains four parts, as shown in Figure 2. The first part is the feature extraction backbone, ResNet50, and its input is a pair of pictures. This part divides the image into the template region pictures and the search region pictures after cropping and data enhancement and sends them to ResNet50 to extract features. The second part is the reconstruction network structure. It reconstructs the extracted features into a vector that the transformer encoder can receive and change as the existing transformer tracker sends the features extracted by ResNet50 to the transformer encoder in element-by-element patches, as shown in Figure 3. This process ensures the integrity of those features to a greater extent. In this part, we use a three-layer reconstruction network structure to enhance the features optimally. Its purpose is to calculate the characteristics of the search region and the template region to form a fusion vector for subsequent classification and regression operations. The fourth part is the predict head which calculates the fusion vector to get the regression bounding box result.

### 3.1. Reconstruction Network

#### 3.1.1. Reconstruction Patch Module

In this work, we reconstructed the feature map of the ResNet output to form vectors that the transformer encoder can accept. The specific operation was a hypothetical feature map $F\in R^{B\times H\times W\times C}$. After the unfold operation $F^{\prime }=Unfold\left(F\right)\;$ was recombined, the calculation for the new patch dimension was as follows (1), (2):

(1) $$L=\prod \frac{\mathrm{feature}\text{\_}\mathrm{size}[d]-1}{stride\left[d\right]}$$

(2) $$Patch_{dim}=\left(B,C\times \prod \left(kernel_{\mathrm{size}}\right),L\right)$$

Therefore, the patch dimension fed into the transformer encoder was $P\in R^{B,C\times \prod \left(kernel_{\mathrm{size}}\right),L}$, which differed from the $P^{\prime }\in R^{B,H\times W,dim}$ patches of other transformer trackers. The patch in our tracker fused the neighbor information of each spatial dimension pixel in the feature map to maximize its integrity.

#### 3.1.2. Transformer Module

After the tracker formed a new patch, it fed it to the transformer for feature enhancement. The patch input in the transformer was multiplied by the Q, K, and V matrices to obtain queries q, keys k, and values v. Then, the transformer encoder performed the attention calculation:

(3) $$Attention\left(Q,K,V\right)=softmax\left(\frac{Q\times K^{T}}{\sqrt{d_{k}}}\right)V$$

The attention calculation does not change the dimension of the input patch, but the spatial dimension of the feature map of the reconstructed patch becomes smaller each time. To ensure the use of the reconstructed patch for subsequent operations, we constructed a new patch. Afterwards, a fully connected layer ensured that the dimension remained unchanged. Therefore, the calculation formula for the entire reconstructed patch module was as follows:

(4) $$O_{reconstrction}=Attention\left(ln\left(Unfold\left(F\right)\right)\right)$$

### 3.2. Cross-Attention

The difference between cross-attention and other attention operations is that the inputs q and kv of cross-attention come from two different branches. Here, we used the last extracted feature of the search region as the source of q in cross-attention and the last extracted feature of the template region as the source of q. The source of K and V is multihead cross-attention, so the calculation formula is as follows:

(5) $$\begin{array}{c} MultiHead\left(Q_{z},K_{x},V_{x}\right)=Concat\left(head_{1},head_{2},\ldots ,head_{n}\right)W^{o}\\ wherehead_{i}=Attention\left(Q_{z}+P_{z},K_{x}+P_{x},V_{x}\right) \end{array}$$

**Figure 4:**
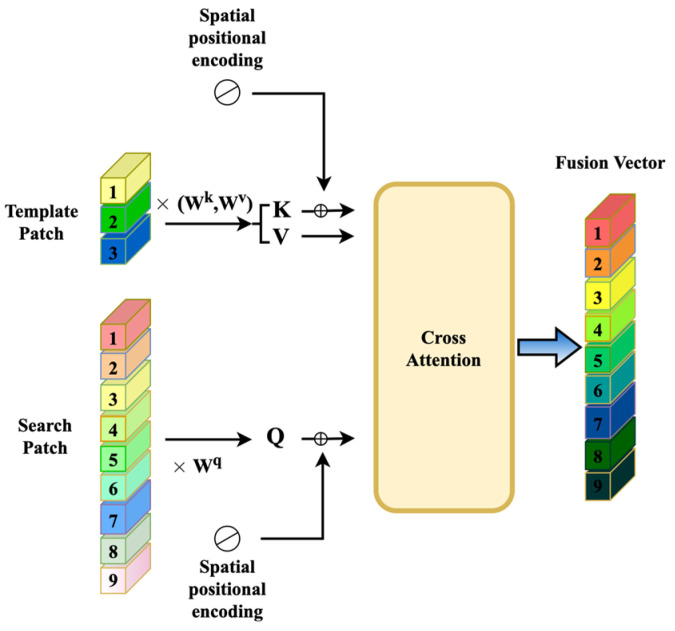
Cross-attention in our method. $\times W^{i}$ means that the input is multiplied by the $W^{i}$ matrix to get i, for example the input is multiplied by $W^{k}$ to get K.

In order to distinguish the position information of the input feature sequence, we referred to the spatial position encoding for the Template patch and the Search patch. Similarly to Transt, we used the sine function to generate spatial position encoding, and only used the position encoding for K and Q, as shown in Figure 4.

### 3.3. Token Performers

As mentioned in [16], the spatial complexity and time complexity of the attention operation are $O\left(N^{2}+Nd\right)$ and $O\left(N^{2}d\right)$, respectively; here, we mainly used fast attention [16], that is, the FA part of the FAVOR+ mechanism to reduce the spatial complexity of the model and, thus, reduce the parameters of the model that have reached the real-time tracking method.

**Figure 5:**
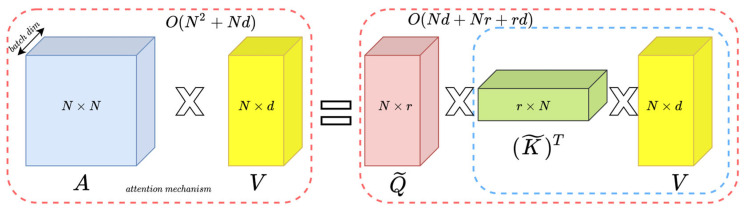
The left side of the figure is the regular attention mechanism, and the right side is the fast attention mechanism. The dashed blocks indicate the order of computation with corresponding spatial complexities attached.

In this work, we used fast attention in the reconstruction network instead of the regular attention mechanism. The specific method is shown in Figure 5. The fast attention calculation formula is as follows:

(6) $$Attention(Q,K,V{)}_{\mathrm{Fast}}=softmax\left(Q\left({\left(K\right)}^{T}V\right)\right)$$

Equation (7) is the spatial complexity of the regular attention mechanism, and Equation (8) is the spatial complexity of the fast attention mechanism.

(7) $$O_{r\;spacial}=O\left(N^{2}+Nd\right)$$

(8) $$O_{F\;spacial}=O\left(Nr+Nd+rd\right)$$

where N is the size of an input sequence of tokens and d is the hidden dimension. Unlike Choromanski et al. [16], r  set the parameters for us, which is one-eighth the size of N. In this work, the spatial complexity when N=64, d=128 was 79% of the regular attention mechanism. As a result, the amount of parameters was reduced.

## 4. Experiment

We used the OTB100 [30], VOT2018 [31], and VOT2021 [32] experiments to analyze and grade the proposed tracking methods. Below, we discuss some experimental setups. Then, we evaluate our model in two of the largest benchmarks OTB100, VOT2018, and VOT2021.

### 4.1. Module Design

Our tracker is trained on a GeForce GTX 3080 GPU using Facebook AI(FAIR) Pytorch1.8.1 [33]. Similarly to SiamRPN, it uses the previous frame as the template frame for template matching. It uses ResNet50 as the base network and initializes the parameters of this network using an ImageNet [34] pretrained model. 

The reconstruction network contains two tiers. To improve the generalization and discriminativeness of our feature representations and avoid overfitting the scarce tracking data, we trained the tracker using the COCO dataset [35], the Lasot dataset [36], the Got-10k dataset [37], and the TrackingNet dataset [38]. They have recently been widely used in tracking methods because they depict many scenes and objects. Our tracker uses Adamw [39] to practice the network from scratch with an initial learning rate of $1\times 10^{-4}$. The model trained for 800 epochs, and the 350th epoch began to reduce the learning rate with a minibatch size of 24. The transformer head was set to 8, and to reduce the number of parameters, the embed dim was set to 128.

### 4.2. Metrics Estimation

We evaluated our approach on two popular and challenging datasets, online tracking benchmark OTB100 and visual tracking baseline VOT2018, VOT2021. OTB100 has 100 real targets for tracking. All the sequences have 11 interference properties. The two standard evaluation indicators of the OTB are success rate and accuracy. For each frame, we calculated the IoU (joint intersection) between the track box and the real bounding box and the distance to their center. One can obtain a success graph by evaluating success rates at different IoU thresholds. Typically, we report the area under the curve (AUC) of the success plot. One can obtain an accuracy map similarly, but accuracy at a threshold of 20 pixels is typical. We used the standard OTB toolkit to get the results. The VOT2018 dataset consists of 60 challenging video sequences. According to the evaluation criteria, the trace fails when the overlap between the estimated and real positions is zero. Then, the tracker is reinitialized to the real position. The metrics used to evaluate VOT datasets included accuracy (A), robustness (R), and expected average overlap (EAO). We define accuracy as the rate at which the estimated location overlaps with ground reality, while robustness is the number of tracking failures. The EAO is a function of sequence length calculated by the average precision of a particular number of frames after the tracker initializes. A good tracker has high A and EAO scores but a lower R score. The VOT2021 dataset consists of 60 challenging video sequences. Unlike the VOT2018 evaluation metric, the robustness (R) of the VOT2021 evaluation metric is the proportion of tracking successful frames. A good tracker has high A, R, and EAO.

### 4.3. Comparison with the State of the Art

Benchmarks including OTB100, VOT2018, and VOT2021 demonstrated the performance of our trackers compared to some of the most advanced technologies. Traditionally, tracking speeds of more than 25 FPS are considered real-time. Our tracker runs at 43 FPS. We obtained all the results in this section using the OTB Toolkit and the VOT toolkit. 

#### 4.3.1. Experiments on OTB100

**Figure 6:**
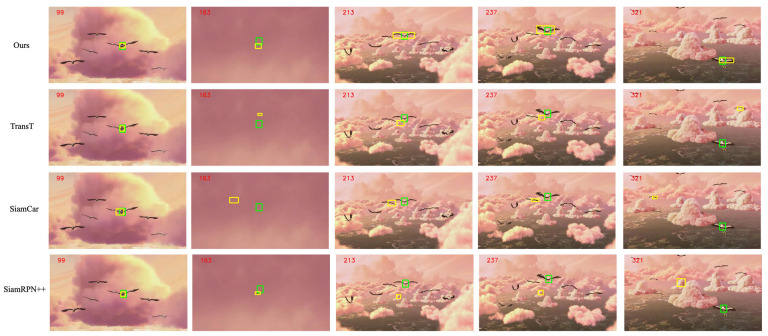
Our method, TransT, SiamCar, and SiamRPN++ in the OTB100 video sequence visualization results, where the number in the upper left corner is the frame of the video sequence, the green rectangle is the real box, and the yellow rectangle is the tracker prediction box.

To verify the effectiveness of our method, Figure 6 shows the visual comparison of our algorithm and the sota algorithm on the OTB100 dataset.

We evaluated the proposed algorithm by comparing it with many of the most advanced trackers: Transt [15], SiamBan, SiamCar, SiamRPN++, SiamFC++, DasiamRPN [40], SiamFC, and Ocean [41].

**Figure 7:**
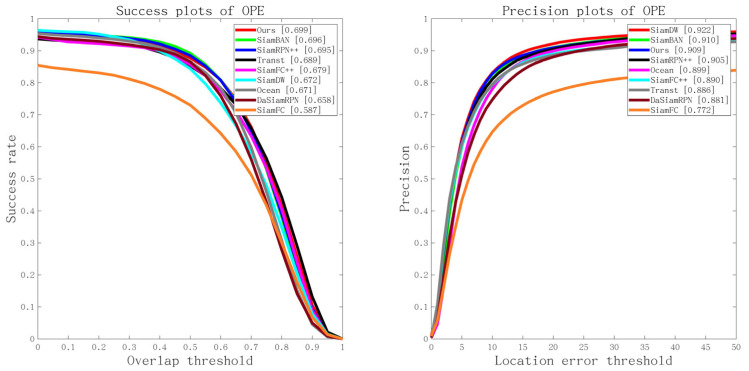
Overall comparison on the OTB100 dataset.

Please note that Transt is the latest sim-based tracker for cross-attention fusion vectors. SiamBan, SiamCar, SiamRPN++, SiamFC++, DasiamRPN, SiamFC, and Ocean are the latest Siam-based trackers for 2D intercorrelation operations. Figure 7 shows the overall performance of our method and other state-of-the-art tracking algorithms in terms of success rates and accuracy plots on OTB-100 [8].

Our method achieved the best results for OTB100 [8], improving the AUC of the second-best tracker SiamRPN by 0.3%. Among the trackers using the Siamese networks, our approach is superior to Transt, SiamBan, SiamCar, SiamRPN++, SiamFC++, DasiamRPN, SiamFC, and Ocean. SiamFC is a groundbreaking tracking framework, but its performance still lags behind recent state-of-the-art methods. Transt was the first tracker to use the transformer mechanism for target tracking, and we still outperformed it by 1%.

#### 4.3.2. Experiments on VOT2018

**Figure 8:**
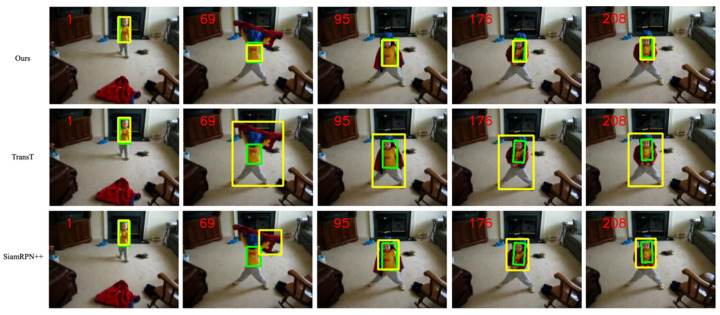
Methods and TransT, SiamRPN++ in the VOT2018 video sequence visualization results, where the number in the upper left corner is the frame of the video sequence, the green rectangle is the real box, and the yellow rectangle is the tracker prediction box.

To verify the effectiveness of our method, Figure 8 shows the visual comparison of our algorithm and the sota algorithm on the VOT2018 dataset.

**Figure 9:**
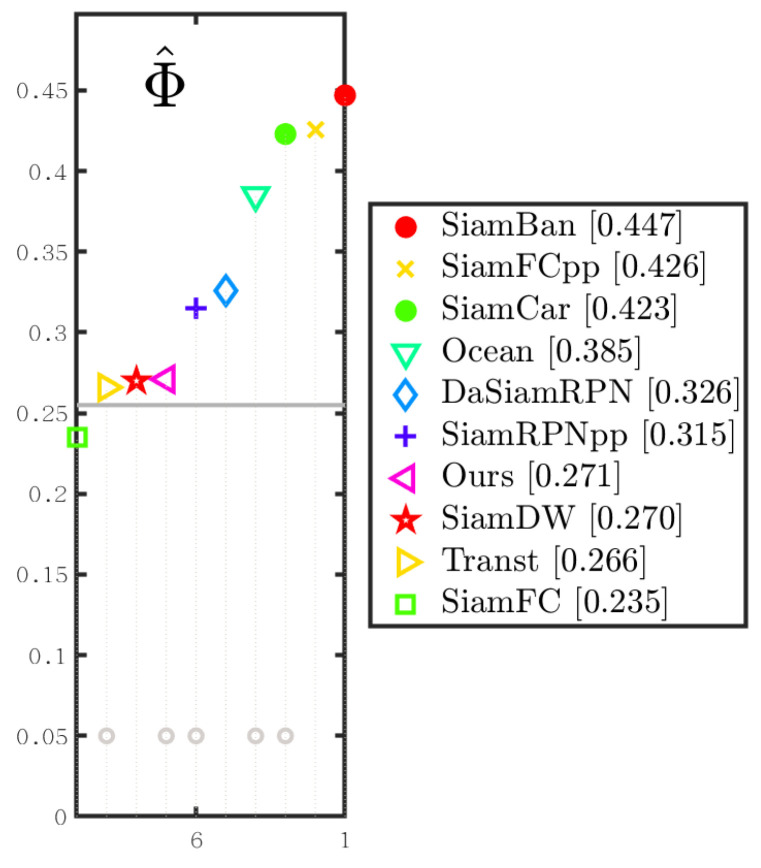
EAO ranking with trackers in VOT2018. The better trackers are located on the right. Best viewed on a color display. $\hat{\Phi }$ is a symbol of a mathematical expression of EAO.

We compared the proposed trackers with nine state-of-the-art tracking algorithms on the VOT2018 dataset: Transt, SiamBan, SiamDW [42], SiamRPN++, SiamFC++, DasiamRPN, SiamDW, SiamFC, and Ocean. We evaluated the proposed methodology at VOT2018 and reported the results in Table 1. As shown in Table 1 and Figure 9, our method achieved an optimal EAO score of 0.271 and an optimal accuracy score of 0.594. Notably, our EAO score and optimal accuracy improved compared to Transt, setting up new and up-to-date technologies that show that our method can significantly reduce tracking failures of the cross-attention’s tracker.

Although our method is at a moderate level of the EAO, it is 1.1% higher than TransT using the cross-attention method.

#### 4.3.3. Experiments on VOT2021

We compared the proposed trackers with seven tracking algorithms on the VOT2021 [32] dataset: SAMN [43], KYS [44], RTT, D3S [45], DiMP [46], PrDiMP [47], ATOM [48]. We evaluated the proposed methodology on VOT2021 and reported the results in Table 2. The algorithmic results mentioned above are all from Kristan et al. [32]. The Refined Transformer Tracker (RTT), a transformer-based tracker that participated in the VOT2021 competition, did not publish papers that only appeared in the VOT2021 competition.

### 4.4. Ablation Experiment

**Figure 10:**
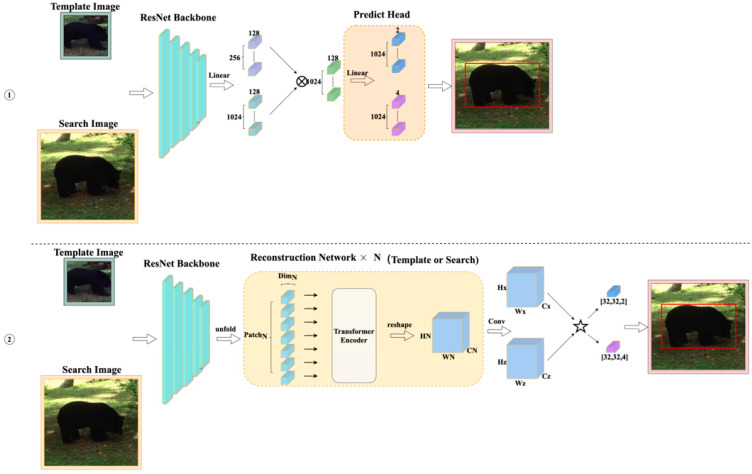
Structure diagram of our ablation experiment.

Comparing our final method in Figure 2, we performed a detailed ablation experiment, and Figure 9 shows our detailed ablation experiment structure diagram. The structure diagram (Figure 10, ①) is a structure without the reconstruction network, and the other is the same as the structure in Figure 2. The structure diagram (Figure 10, ②) replaces cross-attention with cross-correlation (star symbol in the Figure 10, ②), followed by the classification and regression method of SimCar, and the other is the same as the structure in Figure 2. Table 3 gives us the results of the ablation experiment.

## 5. Conclusions

In visual tracking tasks, the matching of templates is critical to the final tracking algorithm for the use of global information and the formation of a new window for the vector after feature extraction. Our paper proposes a tracking network that reconstructs the patch cross-attention method to fuse features and form a fusion patch. We combine the adjacent vectors of feature extraction to form a fusion patch to maximize the use of the feature extracted by the backbone. Then, we use cross-attention to calculate the formed patch, taking advantage of the cross-attention’s long-distance dependence to enhance the accuracy of our method. As can be seen from the experimental results, the proposed technique may be superior to the existing tracking method. In the future, we plan to continue to explore the effective integration of deep networks in tracking tasks.

## Figures and Tables

**Table 1:** Overall comparison for the VOT2018 dataset. ↑ indicate performances ranked at first, ↓ indicate poor performances and - indicate normal performance.

Attributes	Accuracy	Robustness	EAO
SiamRPN++	0.593	0.356	0.315
SiamBan	0.590	0.178	0.447
Transt	0.589	0.384	0.266
Ocean	0.586	0.220	0.385
Siamfc++	0.583	0.173	0.426
SiamCar	0.578	0.197	0.423
SiamDw	0.538	0.398	0.270
Ours	0.594↑	0.487↓	0.271-

**Table 2:** Overall comparison on the VOT2021 dataset.

	Baseline			Real time			Unsupervised
Attributes	EAO	A	R	EAO	A	R	AUC
SAMN	0.457	0.723	0.774	0.439	0.698	0.770	0.537
KYS	0.455	0.724	0.755	0.390	0.684	0.704	0.527
RTT	0.450	0.767	0.727	0.387	0.697	0.696	0.610
D3S	0.443	0.700	0.767	0.432	0.694	0.756	0.505
DiMP	0.432	0.717	0.722	0.415	0.710	0.713	0.588
PrDiMP	0.425	0.724	0.722	0.387	0.691	0.693	0.552
ATOM	0.409	0.711	0.707	0.387	0.707	0.677	0.537
Ours	0.461	0.712	0.787	0.462	0.712	0.788	0.537

**Table 3:** Results of digestion experiments on sequences of OTB100 and VOT2018.

**Attributes**	**OTB100 (Suc** **cess)**	**VOT2018 (acc)**
Ours	0.699	0.594
①	0.643	0.553
②	0.602	0.496
Ours-np	0.684	0.572

## Data Availability

The datasets used in the study are available from the corresponding authors upon reasonable request.
